# Nephron Sparing Surgery for Renal Angiomyolipoma with Inferior Vena Cava Thrombus in Tuberous Sclerosis

**DOI:** 10.1155/2014/285613

**Published:** 2014-02-23

**Authors:** Adrien Riviere, Thomas Bessede, Jean-Jacques Patard

**Affiliations:** Hôpital Bicêtre, 78 Rue du Général Leclerc, 94275 Le Kremlin-Bicêtre, France

## Abstract

*Introduction*. Angiomyolipoma is a common benign renal tumor. It is associated with Tuberous Sclerosis Complex (TSC) in 20% of patients. Angiomyolipomas are classically multiple, bilateral, and growing; they may lead to complications such as Wunderlich syndrome or, in rare cases, to venous extension. *Observation*. a 74-year-old woman with TSC presented with an angiomyolipoma of the right kidney with inferior vena cava (IVC) fatty thrombus. She underwent partial nephrectomy and thrombectomy. After a 7-year follow-up there was no evidence of recurrence or metastasis and her renal function was preserved. *Review of Literature*. It is the 44th reported angiomyolipoma associated with IVC thrombus. The mean size of angiomyolipomas was 86.1 mm and 67.4% of patients were symptomatic. Pulmonary embolism was found in 6 patients. There were 2 cases of recurrence/metastatic outcome after radical nephrectomy and thrombectomy. They were associated with epithelioid form. The mean size of epithelioid tumors was significantly bigger than in classical angiomyolipomas (127.1 mm versus 82.6 mm, *P* = 0.037). With a median follow-up of 12 months, 91.3% of patients were recurrence and metastasis free, with 3 cases of nephron sparing surgery. *Conclusion*. Nephron sparing surgery for angiomyolipoma with IVC fatty thrombus can be safely performed in TSC, even in sporadic angiomyolipoma.

## 1. Introduction

Angiomyolipomas (AMLs) represent 0.3% of all renal tumors. The sex ratio is 4 men for 11 women. Most of the time, AMLs are sporadic but in 20% of patients AMLs are associated with Tuberous Sclerosis Complex. Thus they are classically multiple, bilateral, and growing [1]. They may become symptomatic and may require active management which has to be as conservative as possible. Many cases of inferior vena cava thrombus associated with AML have been reported [2–43]. We report a new clinical case of AML with inferior vena cava thrombus in a TSC patient that raises the question of the best surgical approach regarding the necessity of preserving renal function.

## 2. Materials and Methods

### 2.1. Observation

**Figure 1:**
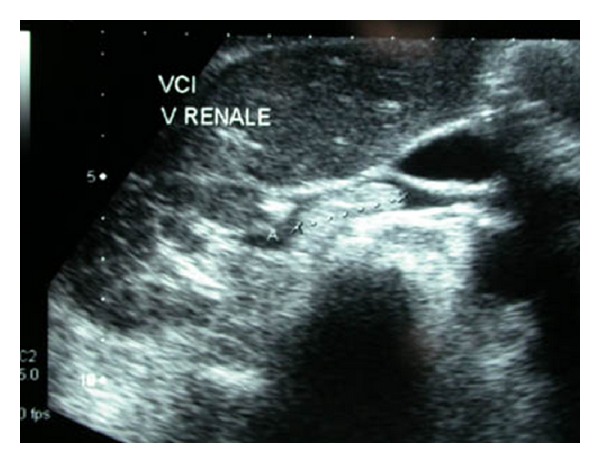
Sonography of the IVC thrombus originated from the right renal vein.

A 74-year-old woman with tuberous sclerosis and multiple bilateral AML who had undergone partial polar superior nephrectomy for a renal cell carcinoma in 1990 came after 7 years of surveillance with the evidence of an inferior vena cava thrombus developed from the right renal vein (Figure 1).

**Figure 2:**
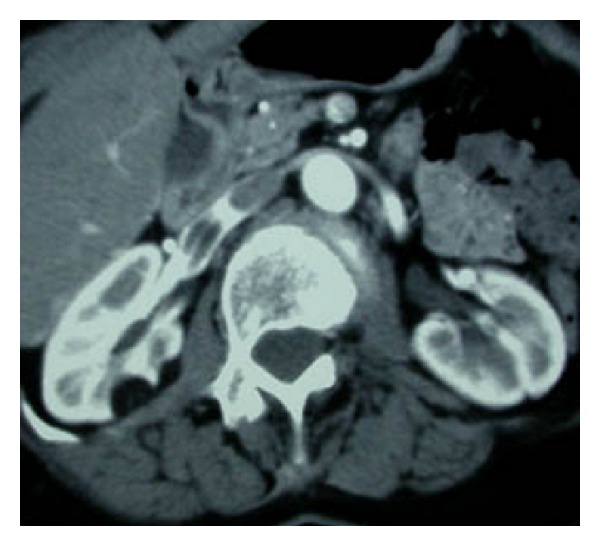
Axial contrast enhanced abdominal CT showing an IVC and renal thrombus with attenuation value—70 HU.

Computerized tomography showed multiple renal tumors with spontaneous density inferior to—20 Hounsfield Units (HU) without contrast enhancement. One of these typical AML that was already present in the previous studies was in contact with a homogenous tumor thrombus, well-circumscribed, with the same fatty density and was extended in the inferior vena cava from the right renal vein, below the hepatic veins (Figure 2). Serum creatinine was 63 *μ*mol/L.

Partial nephrectomy without arterial clamping, lymphadenectomy, or adrenalectomy with thrombectomy was performed. The thrombus was free from the wall of the inferior vena cava and without fibrin clot. The postoperative period revealed to be uneventful. Serum creatinine was 98 *μ*mol/L, on postoperative day 7 when patient was discharged.

Pathologic examination showed a 7-centimeter yellowish tumor, extended with a thrombus presenting the same aspect. It confirmed the diagnosis of AML given by the association of mature adipose tissue, thick-walled blood vessels, and smooth muscle cells. There was no epithelioid contingency.

Clinical and radiological surveillance after 11 years showed no signs of recurrence or metastasis. The renal vein and inferior vena cava were permeable and the renal function was preserved with 78 *μ*mol/L serum creatinine.

### 2.2. Literature Review

A MEDLINE review was performed in order to identify all articles entirely published in English and evaluate inferior vena cava extension of AMLs.

All data are presented in Table 1.

## 3. Discussion

Venous extension of an AML is rare. We have identified 44 cases of inferior vena cava involvement in the literature including this one.

Medium age was 46.6 years (range 16–75 years). It has a clear female predominance, representing 81.9% of patients. It is concordant to what is observed for common AML [1].

AMLs were bilateral in 31.9% of patients but were associated with TSC only in 11.4% of them.

Surprisingly, we found one case, reported by Camúñez et al. [5] with AML and IVC thrombus without right renal vein involvement. The patient had TSC and bilateral AML and did not undergo surgery. A similar case was reported by Ackali et al.; they described a right renal vein thrombus which extended in the IVC without any tumor of the right kidney on ultrasound examination, computer assisted tomography (CT), and even magnetic resonance imaging (MRI) [27]. They concluded that tumor might have originated from the kidney and then extended to the right renal vein and IVC.

Patients were symptomatic in 67.4% of cases; all were experiencing pain. Gross hematuria was present in 9.3% of patients. Other symptoms were nausea and/or vomiting in 9.1% and fever in 6.8%. Five patients (11.4%) presented a Wunderlich syndrome with acute flank pain and drop of blood pressure or hemoglobin with consistent diagnosis of retroperitoneal hemorrhage due to the rupture of the tumor. TSC was present only in one case of retroperitoneal hemorrhage.

We found that the level thrombus reached the diaphragm in 9 patients (20.5%) and got to the right atrium in 6 patients (13.6%).

In this review, almost all tumors were larger than 4 cm with a mean size of 86.1 mm. Only 2 patients presented a small AML with vena cava thrombus; they had TSC. It has been reported that, in TSC, AMLs are more often symptomatic and have a more aggressive growth pattern [44].

As it has been previously reported, there is a large majority of right sided AML with inferior vena cava thrombus (88.6% versus 11.4%). There is no clear explanation for it. According to Islam et al., when thrombus is limited to the renal vein there is no difference between left or right side [25]. In fact, we have to admit that, before extending into the inferior vena cava, the tumor thrombus was in the renal vein and therefore, predominantly, in the right one.

We also highlight the special case of AML with epithelioid cells which are now recognized as an individual tumor, different from classical AML [45, 46]. In this review, recurrence or metastasis was only seen in patients with epithelioid contingency (2 patients, 8.7%). Metastatic localization was the lungs for one and liver and peritoneum for the other. It appears that the size of the tumor is significantly bigger in AML with epithelioid contingency versus classical AML with mean size, respectively, of 127.1 mm versus 82.6 mm (*P* = 0.037, *t*-test).

As reported by Park et al., all reported cases of metastasis of AML in literature were associated with the epithelioid form, expressing the melanocytic marker HMB-45 [28]. The only 2 documented patients with recurrence or metastasis were also epithelioid AML, in our review (HMB-45 positive).

This epithelioid form of AML, characterized by a minor amount of adipose tissues on imaging can mimic the appearance of a clear cell carcinoma [47]. It is reported to quickly evolve towards a metastatic situation with a lethal outcome because of its poor sensitivity to chemotherapy and targeted therapies [48]. Therefore it should be treated aggressively.

There are also malignant tumors presenting with evidence of fat on imaging. Hélénon et al. reported several fat-containing renal cell carcinomas [49]. They were suggesting that diagnosis of AML should be reconsidered in presence of calcification, a large infiltrating or necrotic tumor with association of nonfatty lymph nodes or venous invasion. This review suggests that fat-containing tumors associated with venous fatty thrombus were not malignant tumors at risk of recurrence or metastasis.

In addition, classical AML can be wrongly perceived as clear cell carcinoma in case of recent hemorrhage or spindle cell predominance due to the almost undetectable fat component on imaging [50]. Those cases may benefit from fine-needle biopsy to rule out whether or not the conserving or radical approach should be taken.

Only symptomatic or larger-than-4-centimeter conventional AML should be considered for intervention. Many studies have correlated the risk of hemorrhage/symptomatic presentation with the size of the tumor [51, 52]. In this review, mean tumor size was 86.1 mm. Only several patients had medical history of AML and 11.4% of patients were known to have TSC. Those patients would have benefitted from surgical treatment.

A nephron sparing approach by either selective embolization or open or laparoscopic/robotic partial nephrectomy is recommended when an intervention is required [53–56].

In case of associated venous thrombus, the risk of expansion and cardiopulmonary embolism requires a surgical treatment. Case reported by Shinohara et al. presented with congestive heart failure with a thrombus extended to the right atrium [57].

In case of radical surgery, the prognosis is satisfying. 91.3% of patients remained free from recurrence or metastasis at a median follow-up of 12 months (mean 16.8 months).

Although the presence of a venous thrombus suggests the malignant nature of the primary tumor, conservative surgery is possible. Cases of nephron sparing surgery for T3a or T3b renal cell carcinoma, whether for imperative indications (solitary kidney or renal failure) or intraoperative discovery of the thrombus, showed outcomes that seem acceptable compared to nonconserving surgery [58–60].

## 4. Conclusion

Nephron sparing surgery for AML with inferior vena cava extension in tuberous sclerosis is possible depending on the necessity of renal function preservation. It may be proposed as standard surgery for sporadic AML even with inferior vena cava thrombus.

## Figures and Tables

**Table 1:** 

References	Age	Gender	TSC	Symptoms	Localization	Size (mm)	Thrombus	PE	Fat on imaging	Management	LND	Epithelioid form	AE	Rec	Follow-up (months)
1982 Kutcher et al. [2]	16	f	no	p	Solitary R, upper pole	large	IVC	no	yes	RN, thrombectomy, thoraco-abdominal	no	no	no	nc	nc
1985 Brantley et al. [3]	45	f	no	W: p-N/V-H	Solitary R, upper pole, sinus	90	IVC	no	yes	RN, thrombectomy	no	no	no	no	12
1986 Rothenberg et al. [4]	62	f	no	p-IVD	Solitary L, upper pole, sinus	45	RA	nc	yes	RN, thrombectomy, middline incision, sternotomy	nc	no	no	no	36
1987 Camunez et al. [5]	22	f	yes	W: p-H	Multiple R	small	IVC	no	yes	Follow-up	no	nc	no	no	24
1988 Arenson et al. [6]	22	f	no	p	Multiple R, meRian, sinus	85	IVC	no	yes	RN, thrombectomy	pos	yes	no	nc	nc
1992 Umeyama et al. [7]	75	f	no	p-H	Solitary R, meRian	160	IVC-B	no	yes	NSS and RN, thrombectomy, bi subcostal	no	no	no	no	4
1993 Reiff and Dow [8]	58	f	no	p-Fever	Solitary R, upper pole, sinus	120	IVC	no	yes	RN, thrombectomy, upper transverse, sternotomy	no	no	no	no	6
1993 Honda et al. [9]	58	f	no	no	Solitary R, upper pole, sinus	small	IVC	no	yes	RN, thrombectomy	no	no	no	nc	nc
1994 Morris et al. [10]	58	f	no	p-fever	Solitary R, upper pole	large	IVC	no	yes	RN, thrombectomy, midline, sternotomy	no	nc	no	nc	nc
1994 Moulin et al. [11]	36	f	yes	p	Multiple R, upper pole, sinus	large	IVC	no	yes	RN, thrombectomy	nc	no	no	nc	nc
1995 Leder [12]	30	f	no	p	Solitary R median	large	IVC	no	yes	RN, thrombectomy	nc	nc	nc	nc	nc
1995 Hibi et al. [13]	31	f	no	p-N	Solitary R, lower pole	90	IVC	no	yes	RN, thrombectomy	neg	no	no	nc	nc
1995 Baert et al. [14]	53	f	no	no	Solitary R, upper pole	65	IVC	no	yes	RN and thrombectomy, sub costal	no	no	no	nc	nc
1996 Cittadini et al. [15]	65	f	no	no	Multiple L, sinus	60	IVC	no	yes	Follow-up	no	no	no	nc	12
	67	h	no	W: p-H	Multiple R, meRian, sinus	60	IVC	no	yes	RN, thrombectomy	no	no	no	nc	nc
1997 Rubio- Briones et al. [16]	64	h	no	no	Solitary R, meRian	65	IVC	no	yes	RN, thrombectomy, thoraco abdominal	no	nc	no	nc	nc
1997 Bernstein et al. [17]	45	h	no	no	Solitary R, upper pole, sinus	55	IVC	no	yes	RN, thrombectomy, thoraco abdominal	no	no	no	no	16
1998 Gotoh et al. [18]	52	f	no	p	Solitary R	35	IVC	no	yes	RN, thrombectomy	no	no	no	no	10
1999 Christiano et al. [19]	42	h	no	p-loose of weight	Solitary R, lower pole	205	IVC	no	no	RN, thrombectomy	pos	yes	no	Meta	15
1999 Ito et al. [20]	40	f	no	W: p	Multiple R	large	IVC	no	yes	RN, thrombectomy	no	no	no	no	36
2001 Davydov et al. [21]	46	f	no	p	Solitary R, upper pole	60	RA	no	yes	RN, thrombectomy, middline	no	no	PE	nc	nc
2002 Wilson et al. [22]	69	f	no	no	Solitary R, upper pole, sinus	100	IVC	no	yes	RN, thrombectomy, thoraco abdominal	neg	no	no	nc	nc
2003 Schips et al. [23]	61	f	no	p	Solitary L, upper pole	100	IVC	no	yes	RN, thrombectomy	no	no	no	no	36
2003 Gam$\overset{´}{\text{e}}$ et al. [24]	56	f	no	p	Solitary R, sinus	45	IVC	no	yes	RN, thrombectomy	no	no	no	no	12
2004 Islam et al. [25]	40	f	no	no	Solitary R, Riffuse	110	IVC	no	yes	RN, thrombectomy	nc	no	nc	nc	nc
2006 Haritharan et al. [26]	48	f	no	p-Fever and N/V	Solitary R	150	IVC	no	yes	RN, thrombectomy, bi sub costal, sternotomy	no	no	Hemorrhage	nc	nc
2006 Akcali et al. [27]	55	f	no	p	Solitary R, no renal tumor	0	RA	no	yes	Thombectomy only	no	no	no	nc	nc
2007 Park et al. [28]	69	h	no	nc	Solitary R, Riffuse	130	IVC	no	yes	RN, thrombectomy	nc	yes	no	Rec + Meta	12
2008 Schade et al. [29]	42	f	yes	p	Multiple R, upper pole, sinus	90	IVC	no	yes	RN, thrombectomy, thoraco abdominal	no	no	no	no	22
2008 Ban et al. [30]	70	f	no	no	Solitary R, Riffuse	140	IVC	yes	yes	RN, thrombectomy	no	no	no	no	18
2008 Moudouni et al. [31]	31	f	no	p	Multiple L, diffuse	100	IVC	no	yes	RN, thrombectomy, lombotomy	pos	yes	no	Rec	12
2009 Sandstrom et al. [43]	31	h	no	p-Chest pain	Solitary L, upper pole, sinus	60	IVC	yes	yes	RN, thrombectomy	no	no	nc	nc	nc
2009 Christian and Moon [32]	32	h	no	p	Solitary R, sinus	140	IVC	no	yes	RN, thrombectomy, sub costal	no	no	no	no	2
2009 Durand et al. [33]	57	f	no	no	Multiple R, upper pole, sinus	45	IVC	no	yes	RN, thrombectomy	no	no	no	nc	nc
2010 Tan et al. [34]	44	h	no	no	Solitary R, sinus	100	IVC	no	yes	RN, thrombectomy, bi sub costal	no	no	PE	no	12
2011 Govednik-Horny and Atkins [35]	30	f	yes	no	Multiple R, lower pole	80	IVC	no	yes	Embolization, RN, thrombectomy, lombotomy	no	yes	no	nc	nc
2011 Lopater et al. [36]	34	f	no	no	Multiple R	30	IVC	no	yes	Thrombectomy first then NSS, sub costal	no	no	no	nc	nc
2011 Mittal et al. [37]	46	f	no	p	Solitary R, upper pole, sinus	70	IVC	no	yes	RN, thrombectomy, middline	no	no	no	no	3
2013 Grant et al. [38]	22	f	no	p	Solitary R, Riffuse	90	IVC	no	no	RN, thrombectomy	no	yes	PE	nc	nc
2013 Li et al. [39]	52	f	no	p	Solitary R, lower pole	125	RA	yes	yes	RN, thrombectomy	no	yes	no	no	6
2013 Li et al. [40]	43	f	no	W: p	Solitary R, upper pole	55	IVC	no	yes	Embolization, RN, thrombectomy	no	no	no	no	3
2013 Fernandez-Pello et al. [41]	22	f	no	no	Solitary R, sinus	80	IVC	no	yes	RN, thrombectomy, laparoscopic	no	no	no	no	3
2013 Nouira et al. [42]	34	f	no	p	Multiple R, Riffuse	80	RA	no	yes	RN, thrombectomy, bi sub costal, sternotomy	no	nc	DC sepsis	dc	dc
2013 A. Riviere	74	f	yes	no	Multiple R, meRian	70	IVC	no	yes	NSS, thrombectomy, lombotomy	no	no	no	no	84

R: right; N*∖*V: Nausea*∖*Vomitting; IVC: Inferior Vena Cava; NSS: Nephron Sparing Surgery; nc: not communicated; Meta: Metastasis; L: left; W: Wunderlich syndrome; RA: Right Atrium; PE: Pulmonary Embolism; Pos: Positive; H: gross hematuria; p: pain RN: Radical Nephrectomy; DC: Deseaded; Rec: Recurrence; Neg: Negative; LND: Lymph node Dissection.
